# Proteomics analysis of round and wrinkled pea (*Pisum sativum* L.) seeds during different development periods

**DOI:** 10.1002/pmic.202300363

**Published:** 2024-10-30

**Authors:** Sintayehu D. Daba, Punyatoya Panda, Uma K. Aryal, Alecia M. Kiszonas, Sean M. Finnie, Rebecca J. McGee

**Affiliations:** ^1^ USDA‐ARS Western Wheat & Pulse Quality Laboratory Pullman Washington USA; ^2^ Department of Comparative Pathobiology Purdue University West Lafayette Indiana USA; ^3^ Purdue Proteomics Facility Bindley Bioscience Center Purdue University West Lafayette Indiana USA; ^4^ USDA‐ARS Grain Legume Genetics and Physiology Research Unit Pullman Washington USA

**Keywords:** differentially abundant proteins, label‐free proteomics, protein functional classes, round and wrinkled peas

## Abstract

Seed development is complex, influenced by genetic and environmental factors. Understanding proteome profiles at different seed developmental stages is key to improving seed composition and quality. We used label‐free quantitative proteomics to analyze round and wrinkled pea seeds at five growth stages: 4, 7, 12, 15, and days after anthesis (DAA), and at maturity. Wrinkled peas had lower starch content (30%) compared to round peas (47%–55%). Proteomic analysis identified 3659 protein groups, with 21%–24% shared across growth stages. More proteins were identified during early seed development than at maturity. Statistical analysis found 735 significantly different proteins between wrinkled and round seeds, regardless of the growth stage. The detected proteins were categorized into 31 functional classes, including metabolic enzymes, proteins involved in protein biosynthesis and homeostasis, carbohydrate metabolism, and cell division. Cell division‐related proteins were more abundant in early stages, while storage proteins were more abundant later in seed development. Wrinkled seeds had lower levels of the starch‐branching enzyme (SBEI), which is essential for amylopectin biosynthesis. Seed storage proteins like legumin and albumin (PA2) were more abundant in round peas, whereas vicilin was more prevalent in wrinkled peas. This study enhances our understanding of seed development in round and wrinkled peas.

The study highlighted the seed growth patterns and protein profiles in round and wrinkled peas during seed development. It showed how protein accumulation changed, particularly focusing on proteins implicated in cell division, seed reserve metabolism, as well as storage proteins and protease inhibitors. These findings underscore the crucial role of these proteins in seed development. By linking the proteins identified to *Cameor*‐based pea reference genome, our research can open avenues for deeper investigations into individual proteins, facilitate their practical application in crop improvement, and advance our knowledge of seed development.

**Abbreviations**
AGPas: ADP‐glucose pyrophosphorylase
ANOVA: analysis of variance
DAA: days after anthesis
DAPs: differentially abundant proteins
DW: dry weight
FDR: False discovery rate
FW: fresh weight
GBSSI: granular bound starch synthase 1
PCA: principal component analysis
PSM: Peptide spectral match
SBEI: starch branching enzyme 1
SBEII: starch branching enzyme 2
SSII: soluble starcg synthase 2

## 1 INTRODUCTION

Pea (*Pisum sativum* L.), a staple pulse crop, provides starch (37%–49%), protein (21%–33%), and other nutrients in human and animal diets [1]. Understanding physiological and molecular mechanisms of seed development and protein composition is crucial to increasing agricultural productivity [2]. Seed development is typically divided into three stages: (1) cell division or pre‐storage phase, (2) maturation or storage phase, and (3) desiccation phase [3, 4, 5]. Seed development involves different physiological processes such as seed growth, assimilate transport process, and biosynthetic pathways, each of which regulated by several genes. Cell number is determined by the rate and duration of cell division as well as the number of dividing cells, whereas non‐dividing cell size is determined by cell growth and cell expansion [4]. Matured seeds store protein and non‐protein constituents that enhance germination, provide protection against biotic and abiotic stresses, and provide nutrition for the growing plantlets [6]. In general, to alter and improve seed composition, it is necessary to understand the physiological processes that occur during seed development and the underlying genetic mechanisms.

Mutant and omics resources are crucial for unraveling the molecular mechanisms behind the various physiological processes in pea seed development [7, 8, 9, 10]. Mass spectrometry‐based quantitative proteomic analysis is generally performed using labeled and label‐free techniques [5]. It is generally based on two types of measurements: precursor ion intensity during chromatographic separation or counting of the matched tandem mass spectra (MS/MS counts) of the identified protein [11, 12]. Higher chromatographic peak area/height or the number of proteolytic peptides fragments are always positively correlated with protein abundance, permitting quantitative analysis of the identified peptides/proteins across samples [13]. The label‐free method is a simple but powerful proteomics technology that can be used to gain valuable insights into seed development processes.

Pea seeds are typically classified as round or wrinkled, and these phenotypic differences are the result of the presence and expression of different starch biosynthesis genes [14]. Several pea mutants (lam, r, rb, rug3, rug4, and rug5) that disrupt starch biosynthesis have an impact on seed size and shape as well as seed compositions [15]. These mutants affect *granular bound starch synthase* (*GBSSI*), *starch branching enzyme* (*SBEI*), *ADP‐glucose pyrophosphorylase* (*AGPas*), *plastidial phosphoglucomutase*, *sucrose synthase*, and *soluble starch synthase* (*SSII*), respectively [16]. Proteomics data can reveal important pathways and cellular processes involved in seed development and understanding the specific proteins involved in those pathways that contribute to the phenotypes of round and wrinkled pea seeds. Such information could be crucial for breeding efforts to improve the quality and nutritional value of harvested pea seeds. In this study, we investigated two round peas (one high protein genotype and one low protein genotype) and one wrinkled pea. The objective of this study was to assess the proteome profile differences during seed development period of the high and low protein pea genotypes as well as the proteome profile differences between the wrinkled and round pea genotypes.

## 2 MATERIAL AND METHODS

### 2.1 Plant materials and sampling

Three pea genotypes were used in the study: two smooth‐seeded types, *Cameor* (designated E1) and *PS17100006* (designated E4), and one wrinkled‐seeded type, *PI 357292* (designated E3). *Cameor* is a high‐protein cultivar, while *PS17100006* is a low‐protein breeding line. *PI 357292*, sourced from the USDA‐ARS germplasm repository (https://www.ars‐grin.gov/), has a wrinkled seed surface and a high protein content. Images of seeds of the three genotypes are shown in Figure S1. For each genotype, two seeds were sown in 3.8 L pots in a greenhouse in Pullman, Washington. After germination, approximately 2 weeks post‐planting, the seedlings were thinned to one per pot. To ensure sufficient pods for all sampling time points, 28 pots were prepared for each entry. Pots were filled with Sunshine Mix #1 (SunGro Horticulture, Agawam, MA, USA) and irrigated daily throughout the growth period. Approximately 20 g of Osmocote (14‐14‐14 NPK, Everris International B.V., Dublin, OH, USA) were applied 2 weeks after planting.

Pea flowers were tagged with the date of anthesis to track pod development. Pods were collected at 4, 7, 12, and 15 days after anthesis (DAA), and at physiological maturity. Physiological maturity was defined as when pods dried and turned straw‐colored, typically around 37 DAA, and is referred to as maturity in this study. For each sampling time point, four replicate samples were taken. Samples were collected in either 15 or 50 mL centrifuge tubes and flash‐frozen in liquid nitrogen. They were then stored at −80°C until seeds were extracted for proteomic analysis. Sufficient seed samples from each time point were packaged in dry ice and sent to the Purdue University Proteomics Facility, West Lafayette, Indiana for analysis. Additionally, samples were collected at 7, 12, 15, 18, 21, 24, 27, 32, and 37 DAA to measure fresh and dry weights (FW and DW) as well as moisture content during seed development. The remaining seeds from each genotype were harvested separately to determine protein and starch contents. Protein content (N x 6.25%) was measured using an FP828p protein analyzer (LECO Corporation, St. Joseph, MI, USA). Starch content (%) was determined using the Megazyme starch test kit following AACC Method 76‐13.01 [17].

### 2.2 Sample preparation and LC‐MS/MS analysis

Frozen pea seeds were wrapped in aluminum foil, crushed with a mortar and pestle, transferred to bead rupture Precellys CK28‐R tube (Bertin Corp., Rockville, MD, USA) with lysis buffer (5% sodium dodecyl sulfate [SDS], 50 mM triethylammonium bicarbonate [TEAB] pH 8.5). Samples were then homogenized in a hard tissue homogenizer six times with 3 × 20 s in each cycle. The lysates were transferred to new tubes and bath sonicated for 30 min and centrifuged at 5000 x *g* for 5 min at 4°C to get rid of the debris. The protein content was measured using the bicinchoninic acid (BCA) assay with BSA as a standard. For each sample, 50 µg (or equivalent volume) of protein was taken, and TEAB buffer was added to the samples to adjust to equal volumes across all samples. The samples were reduced with 5 mM tris(2‐carboxyethyl) phosphine (TCEP), incubated at 55°C for 15 min, followed by alkylation with 20 mM methyl‐methanethiosulfonate (MMTS) and incubated in the dark for 10 min. The samples were acidified with 2.5% phosphoric acid to completely denature the proteins, followed by addition of 165 µL of binding/wash buffer (100 mM TEAB in 90% methanol) and mixed immediately. These were passed through the S‐Trap micro spin columns (Protifi, USA) and centrifuged at 2000 x *g* for 1 min. The S‐trap columns were washed three times with the binding/wash buffer with centrifugation at 2000 x *g*. These columns were then transferred to clean 1.7 mL Eppendorf tubes and the trapped proteins were digested overnight with Pierce Trypsin Protease, MS Grade (ThermoFisher Scientific, USA) at 1:25 (enzyme:substrate ratio) in a thermomixer at 37°C. Trypsin was resuspended in 50 mM TEAB buffer. Purified peptides were eluted using 50% acetonitrile, 50 mM TEAB, and 0.2% formic acid via centrifugation for 1 min at 2000 x *g*. They were subsequently dried in a vacuum centrifuge heated to 45°C.

Dried purified peptides were resuspended in 3% ACN, 0.1% FA in water to a final content of 1 µg/µL and 1 µL of peptides was loaded into Thermo PepMap Neo trap column (5 µm long, 300 µm x 5 mm, 1500 bar) at a flow rate of 10 µL/min and then, separated by reverse phase using an IonOpticks Aurora Ultimate analytical columns (25 cm x 75 µm with pore size 120 Å and particle size 1.7 µm) in the Dionex Ultimate 3000 HPLC coupled with Orbitrap Fusion Lumos Tribrid (Thermo Fisher Scientific) mass spectrometer. The peptides were separated using a 130‐min mobile phase gradient with a flow rate of 400 nL/min. The mobile phases A comprised 0.1% FA in water and B comprised80% ACN, 0.1% FA in water. For the first 5 min, the gradient of B was maintained at 2%, and increased to 8% B after 5 min. Then, the percentage of B was linearly increased to 27% till the 80 min. The gradient of B was linearly increased to 45% for the next 20 min, then increased to 100% and kept constant for the next 12 min, and finally decreased to 2% and maintained at 2% B for the last 8 min. Mass spectrometry analysis was conducted using the Orbitrap Fusion Lumos Tribrid Mass Spectrometer. MS1 was acquired using an Orbitrap with a resolution of 60,000 and a scan range of 350–1600 *m*/*z*. MS/MS was acquired using an HCD with a collision energy of 30%, resolution of 15,000, and isolation window of 1.2 *m*/*z*. LC‐MS/MS data were collected using four biological replicates per treatment condition for statistical analysis.

### 2.3 Database searching for protein identification

The raw MS/MS data were searched using MaxQuant version 2.0.3.0 against the *P. sativum* high protein cultivar *Cameor* reference genome database that has 57,835 protein sequences [9]. The following search criteria were modified: trypsin/P enzyme selectivity permitting up to two missed cleavages; oxidation of methionine (M) as a variable modification, alkylation at cysteine residues was set as fixed modification using MMTS and precursor mass tolerance was set to 10 ppm. Peptide spectral match (PSM) and protein identification's false discovery rate (FDR) was set at 1%. For peptide quantification, non‐redundant, non‐unique peptides assigned to the protein group with most other peptides—the unique plus razor peptides—were employed. Intensity measurements from label‐free quantification, or LFQ, were utilized to calculate relative protein abundance.

### 2.4 Data analysis and visualization

Venn diagrams [18] were generated for each pea genotype across the five growth stages to examine both shared and unique proteins at different seed growth stages. Proteins with significantly different abundances (*p* < 0.01) across genotypes, growth stages, and their interaction were identified using analysis of variance (ANOVA) with Perseus v1.6.0.9 [19]. Principal component analysis (PCA) was performed on all detected protein groups to assess data patterns and variability. The first two principal components were plotted in two ways: (1) considering growth stages as the grouping variable and (2) considering a combination of genotypes and growth stages as the grouping variable. PCA was critical for evaluating the reproducibility of the quantification procedure and the reliability of the data for further analyses. Additionally, cluster analyses were performed separately on protein groups that showed significant differences (*p* < 0.01) for lines and growth stages. These analyses helped to elucidate distinct patterns in protein abundance across the different experimental conditions.

Differentially abundant proteins (DAPs) were identified based on two criteria: a *q*‐value ≤0.01 and logFC ≥0.58 or ≤ −0.58. These analyses were performed in a pair‐wise comparison among three growth stages (7 DAA, 15 DAA, and maturity) across the three pea genotypes. The results were visualized using volcano plots, which highlight the significance and magnitude of protein abundance changes.

Protein functional characterization was performed using the Mercator4v6.0 protein classification and annotation framework [20]. This tool allowed us to categorize and annotate proteins based on their functions. We also examined storage proteins, as reported by Kreplak et al. [9], and starch biosynthesis enzymes, as documented by Yu et al. [15]. Additionally, Mercator4v6.0 provided access to protein descriptions from prot‐scriber and swissprot databases, enriching our understanding of the functional roles of the identified DAPs.

## 3 RESULTS

### 3.1 Seed growth and compositions

**FIGURE 1:**
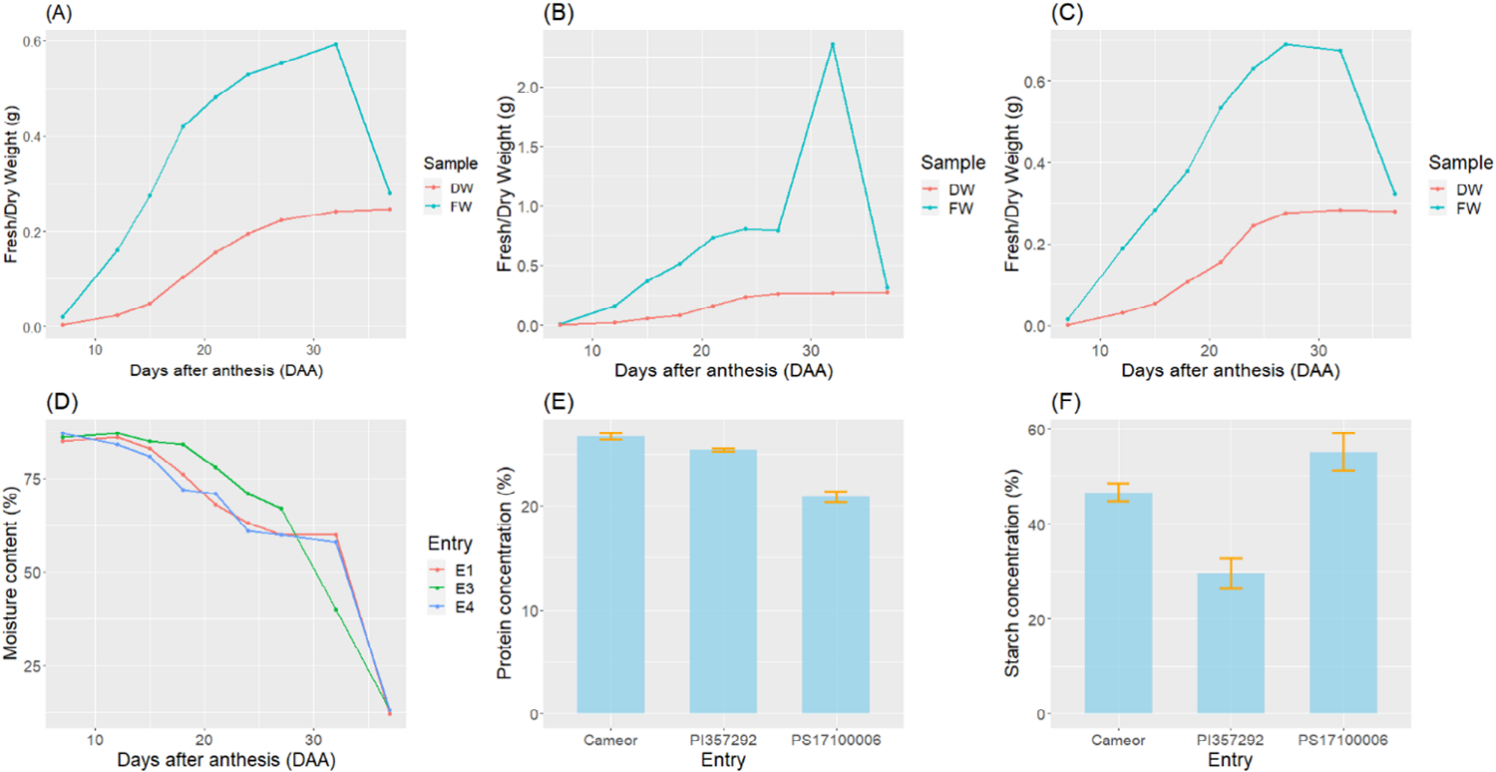
Fresh and dry weight changes during seed growth period for (A) E1: *Cameor*, (B) E3: *PI 357292*, and (C) E4: *PS17100006*, (D) moisture content changes during seed growth period, (E) protein content of mature seed, and (F) starch content of mature seed.

The changes in FW, DW, and moisture during the seed growth period are illustrated in Figure 1. The FW of the seeds increased steadily until 32 DAA for entry E1 (*Cameor*) and until 27 DAA for entry E4 (*PS17100006*), after which it began to decline (Figure 1A, B). According to Stickland and Wilson [21], the maximum FW in peas is typically reached around 1 month after flowering. In contrast to the round pea entries (E1 and E4), the wrinkled pea entry E3 (*PI 357292*) showed a different FW growth pattern. For E3, a significant FW increase occurred between 27 and 32 DAA (Figure 1C), primarily due to higher seed moisture content. Across all three genotypes, there was a notable increase in DW between 15 and 27 DAA. Throughout most growth stages, there was a significant difference between FW and DW, except at maturity. By approximately 37 DAA, when the genotypes were physiologically mature and the seed moisture content was 12%–13%, the difference between FW and DW was minimal. Initially, at 4 DAA, seed moisture was over 85% of the FW, but it progressively decreased throughout the seed growth period. Rapid seed drying began around 32 DAA for all entries (Figure 1D).

The mean protein content for *Cameor*, a smooth pea, was 26.7% ± 0.29% (Figure 1E). The other smooth pea, *PS17100006*, had a lower protein content with a mean of 20.9% ± 0.18%. Wrinkled peas typically have high protein and low starch contents [14], a trend consistent with our findings. The wrinkled pea (*PI 357292*) in our study had a high mean protein content (25.4% ± 0.56%) and a low starch content (29.5% ± 3.2%) (Figure 1E, F). As anticipated, the low‐protein genotype (*PS17100006*) had the highest starch content among the three lines, at 55.1% ± 3.9%. *Cameor* followed with a starch content of 46.6% ± 2.0%. The starch content for the wrinkled pea genotype (*PI 357292*) was the lowest, at 29.5% ± 1.6%.

### 3.2 Overview of the proteomics profiles in three pea genotypes

A search of the *Cameor*‐based pea reference genome [9] resulted in a total of 3659 protein groups across all 15 samples (Table S1). The number of pea gene variants in the protein groups ranged from 1 to 27, with an average of 2 per protein group. Multiple sample test showed that 3026 protein groups were found to be significant a *q* ≤ 0.01 (Table S2). The number of significantly different (*p* ≤ 0.01) protein groups in the comparisons of the genotypes, growth stages, and both were 735 (Table S3), 2717 (Table S4), and 1119 (Table S5), respectively.

**FIGURE 2:**
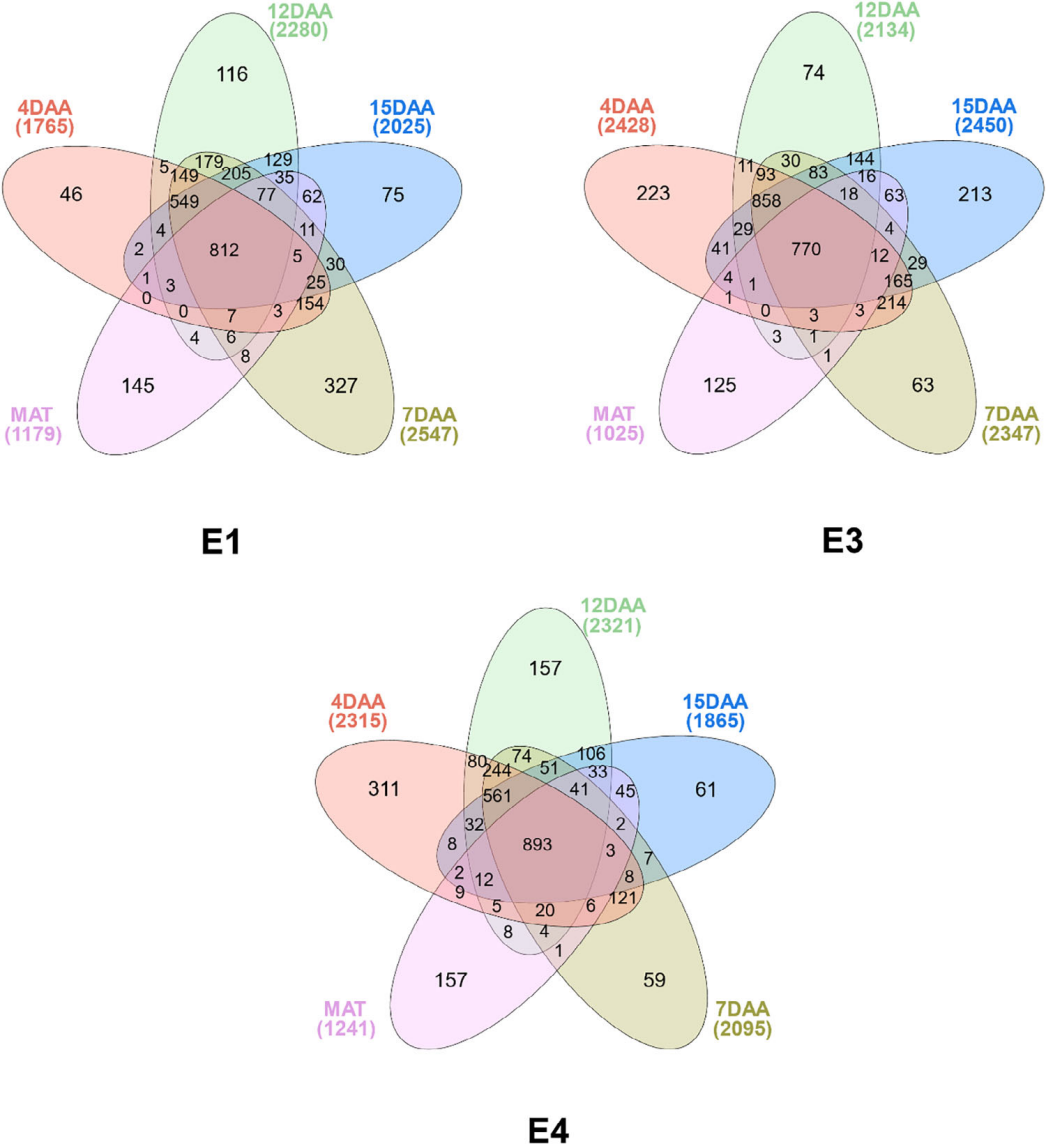
Venn diagrams that show the number of common and unique protein groups detected at each growth period for (E1) *Cameor*, (E3) *PI 357292*, and (E4) *PS17100006*.

More protein groups were detected in the first 15 DAA compared to mature seeds (Figure 2). The highest number of protein groups was observed at different times for each genotype: at 7 DAA for E1 with 2547 protein groups, at 12 DAA for E4 with 2321 protein groups, and at 15 DAA for E3 with 2450 protein groups. In contrast, the lowest number of protein groups for each genotype was detected at maturity, with protein group counts of 1179 for E1, 1025 for E3, and 1241 for E4. Neto et al. [22] similarly reported fewer proteins in the late seed development stages of acai palm seeds, attributing this to the presence of high‐abundance proteins and the challenge of extracting proteins from thick, lignified tissues. Protein groups that were shared among all the time‐points ranged in number from 770 for E3 to 893 for E4 (about 21%–24% of the detected protein groups). Unique protein groups at specific time‐points were 46–327, 63–223, and 59–311 for E1, E3, and E4, respectively. The most unique protein groups were detected at 4 DAA for E3 (*n* = 223) and E4 (*n* = 311), and 7 DAA for E1 (*n* = 327). At maturity, 125–157 protein groups were found to be unique for the three entries.

**FIGURE 3:**
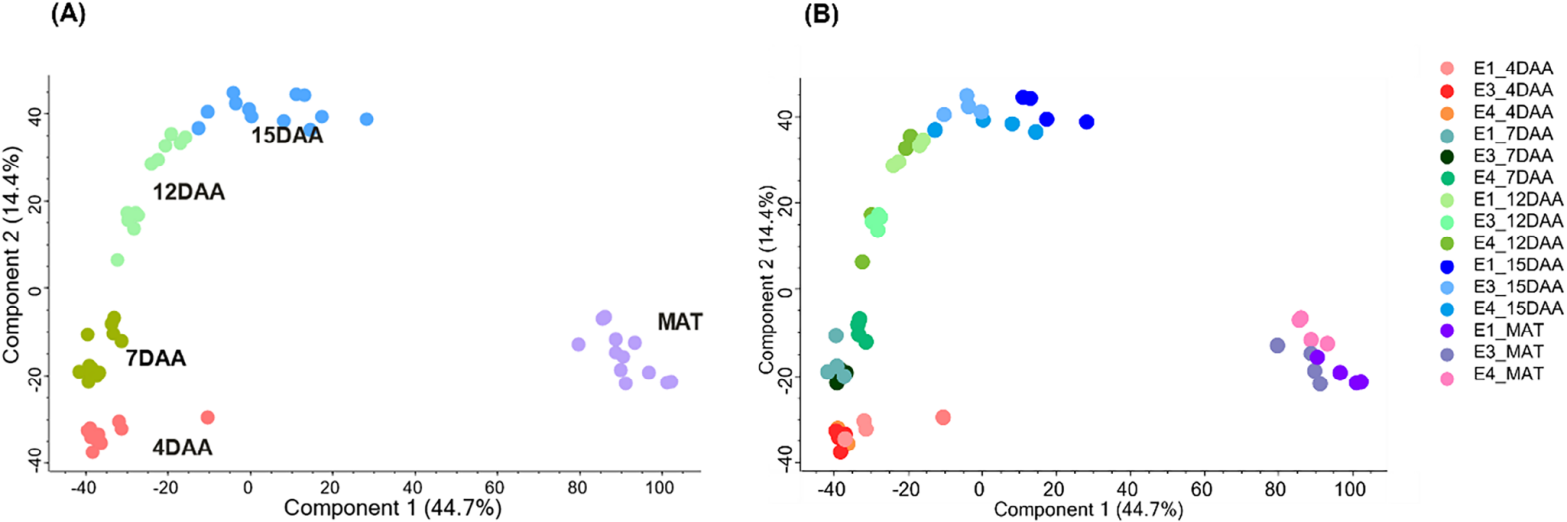
Principal component analysis (PCA) plots showing replicate‐to‐replicate variation (A) for seed growth stages and (B) for entries within each growth stage. E1 = *Cameor*, E3 = *P I357292*, and E4 = *PS17100006*.

The first two principal components accounted for 59.1% of the total variation (Figure 3). Overall, the samples were discriminated according to time‐points, in which the first principal component clearly separated maturity from the early growth stages (Figure 3A). The second principal component further separated 4 and 7 DAA from 12 and 15 DAA. Within each growth stage, the entries were shown to have different proteome patterns (Figure 3B). For example, E1 and E3 had a closer proteome pattern as compared to E4 at 7 DAA. In general, E3 had a different proteome pattern as compared to E1 and E4 at 12 DAA. Even if the entries had closer proteome pattern at 4 DAA, replicate 1 for E1 was an obvious outlier.

**FIGURE 4:**
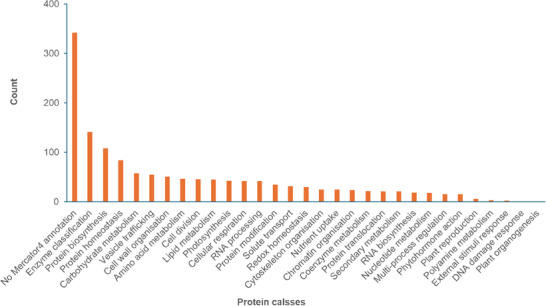
Functional classifications for 1422 of the proteins identified across the 15 samples (three lines by five growth stages).

Using Mercator4 v.6 [20], we classified 1080 pea proteins into 30 functional classes, with each class containing between 1 and 141 proteins (Figure 4). The largest category was enzyme classification, encompassing 141 proteins. This was followed by *protein biosynthesis* with 108 proteins, *protein homeostasis* with 84 proteins, *carbohydrate metabolism* with 58 proteins, *vesicle trafficking* with 55 proteins, and *cell wall organization* with 51 proteins. Additionally, several other classes contained more than 40 proteins each, including *amino acid metabolism*, *cell division*, *lipid metabolism*, *photosynthesis*, *cellular respiration*, and *RNA processing*. While 335 pea genes did not have annotations according to Mercator4v.6, they were categorized using prot‐scriber and swissprot databases (Table S6).

**FIGURE 5:**
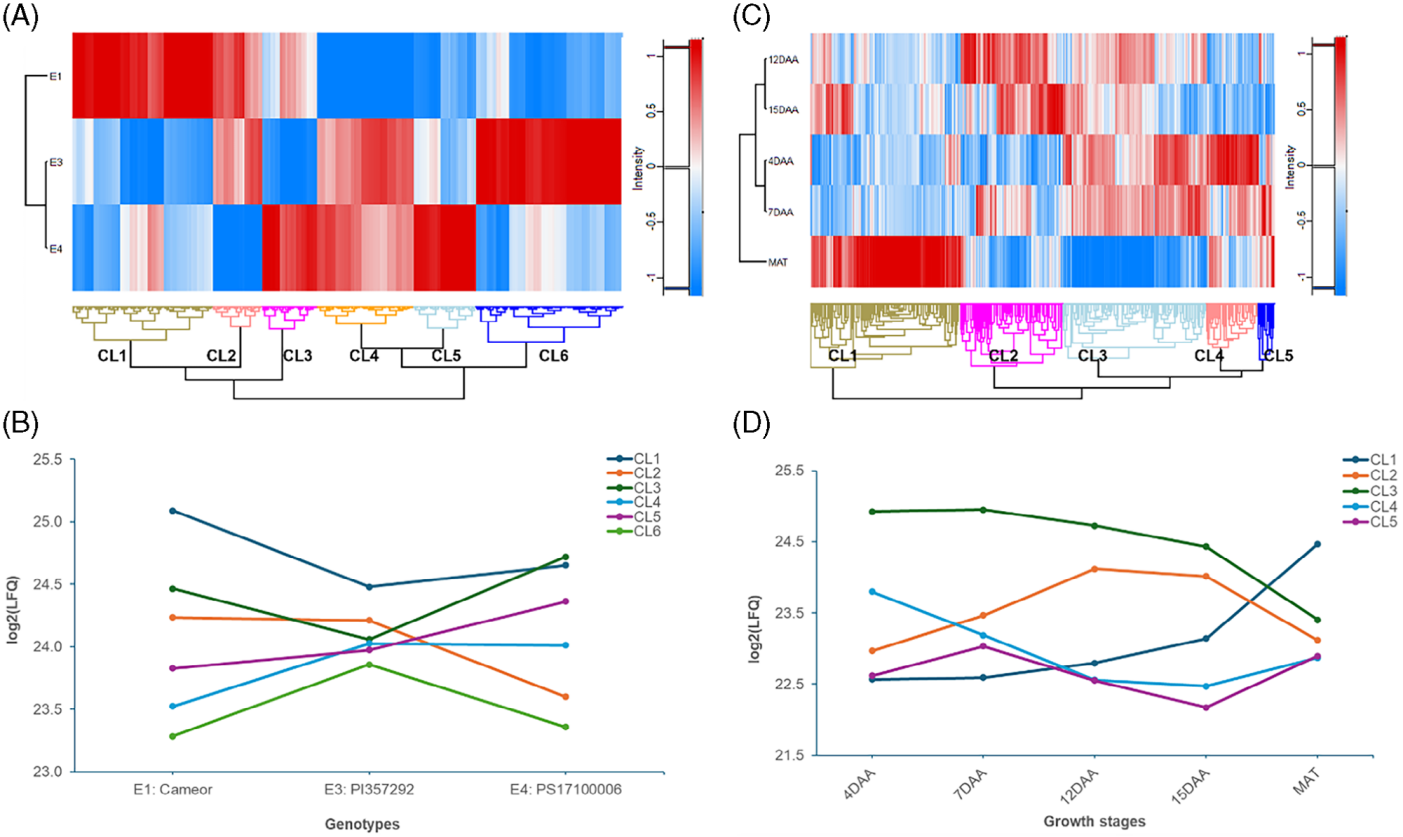
Hierarchical clustering with heat map of proteins significant among genotypes (A) and growth stages (B) along with the average changes in protein abundance in cluster across genotypes (C) and growth stages (D).

Hierarchical cluster analysis revealed six clusters (CL1–CL6) for proteins significant among the three genotypes (Figure 5A), with the number of protein groups ranging from 66 in CL2 to 197 in CL6. The average protein abundance in each cluster with respect to the three lines is given in Figure 5B. For instance, the abundance proteins in CL1 and CL3 were generally found to be higher on average for *Cameor* and lower for *PI 357292*. Clustering of proteins significant across the five growth stages resulted in five clusters (Figure 5C), with the number of protein groups ranging from 97 in CL5 to 879 in CL1. The growth stages are generally grouped in three clusters: with 12 and 15 DAA in the first cluster; 4 and 7 DAA in the second cluster; and maturity separately in the third cluster. The trends in protein abundance across the growth stages in the five clusters are given in Figure 5D. Proteins in CL1 showed an increase while those in CL3 showed a decrease abundance as the growth stages advanced. CL2 proteins increased to 12 or 15 DAA and then decreased at maturity. Proteins in CL4 decreased in the first 12 DAA and then stabilized or increased slightly. CL5 proteins increased at 7 DAA, then deceased to 15 DAA, and finally increased at maturity.

### 3.3 Functional characterization of proteins

Using Mercator4v6.0 [20], we grouped 1080 of the 3659 protein groups into 31 functional classes (Table S6). Additionally, we found descriptions for 335 proteins using *prot‐scriber* and *swissprot*. Our study identified 13 of the 26 pea genes involved in starch production as reported by Yu et al. [15]. Five of these had different annotations with Mercator4v6.0, while eight had no Mercator4v6.0 annotation. According to Kreplak et al. [9], the pea genome has 40 storage protein genes, coding for *albumin*, *legumin*, *vicilin*, and *convicilin*. We identified 14 of these storage proteins in our study, none of which had Mercator4v6.0 annotations. Overall, 1437 proteins from our study have either annotations or descriptions, and some of these categorized proteins are discussed in subsequent sections.

#### 3.3.1 Cell division

Cell division during early seed development is crucial for determining seed size [3]. Genes regulating this process are vital for seed development. In our study, we detected 46 proteins related to cell division using Mercator4v6.0 [20]. These were classified into five categories: *cytokinesis* (28 proteins), *cell cycle organization* (10 proteins), *mitochondrion/peroxisome binary fission* (five proteins), *DNA replication* (two proteins), and *plastid division* (one protein) (Table S6). These proteins are encoded by 23 different pea genes.

Six of the variants of *Psat1g019120* that code for cell plate maturation factor *(AIR9) were found to be significant (*p* = 4.94 × 10^−7^) among the three genotypes (Table S2). The highest abundance for this protein was found in the wrinkled pea genotype *PI 357292*, followed by *Cameor* and the lowest for *PS1710006*. A total of 34 cell division related proteins encoded by variants of 15 pea genes (*Psat1g164200*, *Psat2g132120*, *Psat2g132080*, *Psat2g023680*, *Psat1g019120*, *Psat2g018320*, *Psat1g169360*, *Psat2g009880*, *Psat2g116080*, *Psat1g039040*, *Psat1g091400*, *Psat2g019240*, *Psat1g213760*, *Psat2g015800*, and *Psat1g032800*) were found to be significantly different across the five growth stages (Table S3). Proteins such as ER‐tubule curvature‐inducing protein *(Reticulon), microtubule‐associated protein *(MAP65‐2), component *(RFC3) of PCNA sliding clamp loader complex, dynamin‐like protein *(DRP3), fission factor *(PMD), and regulatory protein *(FRIENDLY) of mitochondrion division were more abundant at early seed growth stage (4 or 7 DAA) and decreased during the seed development period. Some others, for example, component *(INCENP/WYRD) of CPC chromosome passenger complex and cell plate maturation factor *(AIR9) were more abundant at maturity. Some of the proteins showed different patterns of proteomic profiles among the three genotypes. One example of such a protein is catalytic component *(CDKA) of cyclin‐dependent kinase complex, which was more abundant at 4 DAA in Cameor while it was more abundant at maturity in the other two genotypes.

#### 3.3.2 Photosynthesis and seed reserve metabolisms

##### Photosynthesis‐related proteins

Chlorophyll‐containing, immature green seeds can perform photosynthesis, providing energy for embryo growth [23]. In the current study, 43 proteins involved in photosynthesis (Table S6), categorized further as *photophosphorylation* (22 proteins), *Calvin cycle* (nine proteins), *photorespiration* (eight proteins), and *CAM/C4 photosynthesis* (four proteins). Proteins related to *photophosphorylation* and the *Calvin cycle* predominantly accumulated at 12 DAA. Those involved in *photorespiration* peaked at 4 DAA, while *CAM/C4 photosynthesis‐related* proteins were more prevalent at 7 or 12 DAA.

##### Carbohydrate metabolism

A total of 58 proteins involved in carbohydrate metabolism were identified as annotated by Mercator4v.6.0 [20], classified further into nine subclasses including nucleotide sugar biosynthesis (20 proteins), sucrose metabolism (17 proteins), and starch metabolism (eight proteins) (Table S6). The 58 proteins are encoded by variants of 36 pea genes.

Among the three genotypes, 10 of these proteins, regulated by variants of six pea genes, showed significant differences (*p* < 0.01). *Alpha‐galactosidase* (*AGAL*) and *the small subunit (APS) of ADP‐glucose pyrophosphorylase*, as well as *sucrose synthase* controlled by variants of *Psat1g118240*, *Psat2g005160*, and *Psat1g139760*, were more abundant in the high‐protein genotype *Cameor*. *Ribokinase* and *cytosolic UDP‐glucose pyrophosphorylase* encoded by variants of *Psat1g007800* and *Psat2g043240* were more abundant in the low‐protein genotype *PS1710006*. *Pyruvate decarboxylase (PDC)*, encoded by one variant of *Psat1g199560*, was found to be more abundant in the wrinkled pea genotype *PI 357292*.

Across the growth stages, 52 out of 58 proteins showed significant differences at *p* < 0.01, with 17 related to *sucrose biosynthesis* and 7 to *starch biosynthesis* (Table S6). The 52 proteins were encoded by variants of 30 pea genes. *Sucrose synthase* and *starch synthase (SS2)*, regulated by *Psat1g139760* and *Psat1g073520* respectively, were more abundant at 12 or 15 DAA across all entries, with lower levels at maturity for the former and at 4 DAA for the latter. *Sucrose synthase* catalyzes the reversible cleavage of *sucrose* into *fructose* and *uridine diphosphate glucose (UDG‐G)* or *adenosine diphosphate glucose (ADP‐G)*, crucial for energy production, and complex carbohydrate synthesis like starch [15, 24]. *AGAL*, encoded by *Psat1g118240*, steadily increased during seed development, with the highest at maturity (Table S6), consistent with acid *α‐galactosidase* expression in peas as reported by Blöchl et al. [25].

**FIGURE 6:**
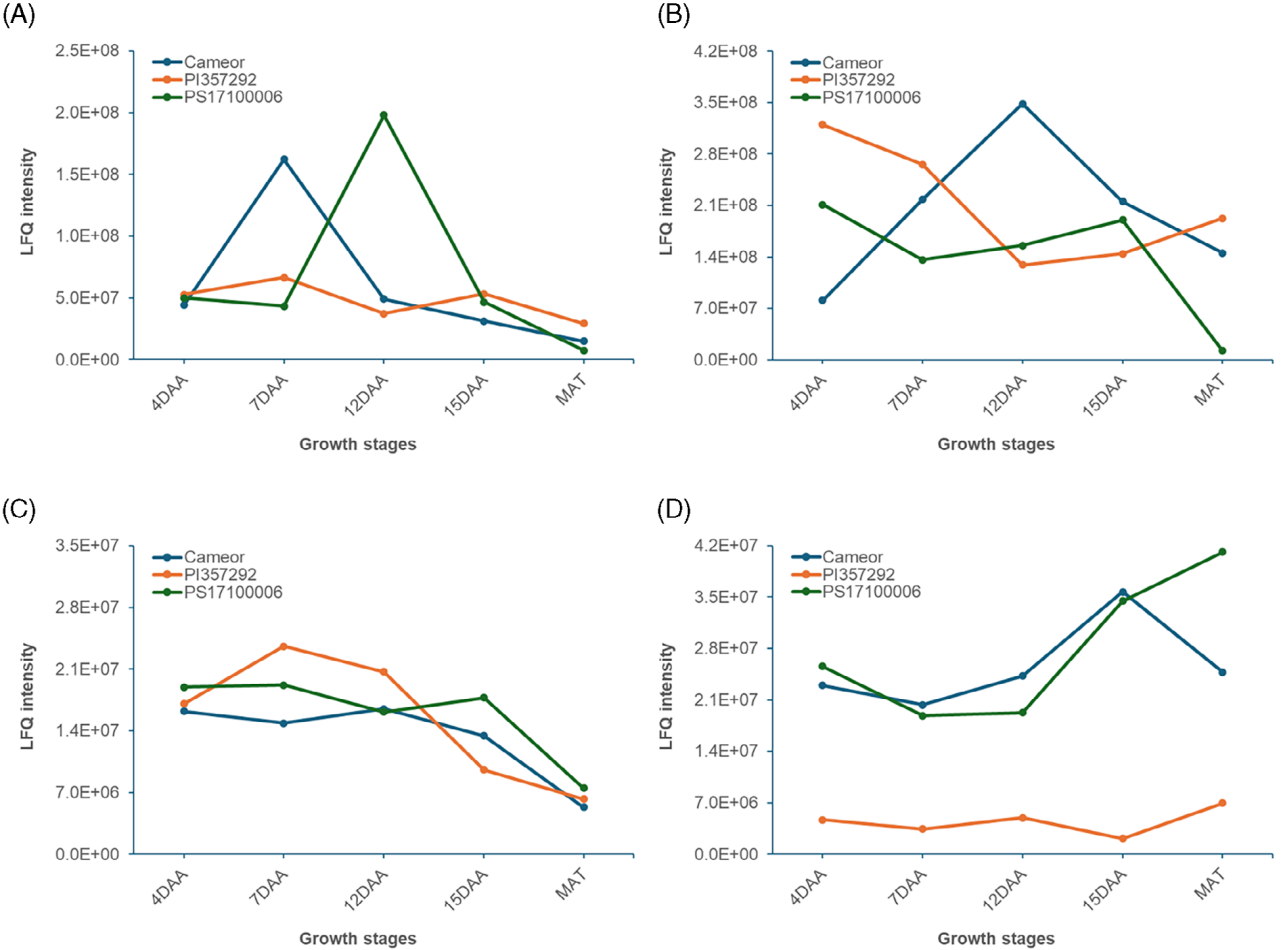
Relative protein abundance (LFQ) during the seed growth period for (A) *GBSSIb* encoded by *Psat0s3525g0120*, (B) *SSII* encoded by *Psat1g071360*, (C) *SSIII* encoded by *Psat1g178480*, and (D) *SBEI* encoded by *Psat3g034640*.

Yu et al. [15] identified 23 pea genes involved in starch biosynthesis. In our study, we found 13 of these genes (*Psat5g006240.1*, *Psat5g110320.1*, *Psat2g004680.1*, *Psat0s425g0240.1*, *Psat0s3525g0120.1*, *Psat5g049640.1*, *Psat6g201440.1*, *Psat3g034640.1*, *Psat1g071360.1*, *Psat1g178480.1*, *Psat4g019040.1*, *Psat1g139440.1*, and *Psat2g042520.1*) encoding enzymes such as *AGPas_L1*, *AGPas_S1*, *AGPas_S2*, *GBSSIa*, *GBSSIb*, *ISA3*, *PGM*, *SBEI*, *SSII*, *SSIII*, *SuSy1*, *SuSy3*, and *UGPase* (Table S6). Overall, these starch biosynthesis enzymes were more abundant in the low‐protein genotype (*PS17100006*), followed by the high‐protein genotype (*Cameor*), and the wrinkled pea genotype (*PI 357292*) with the least abundance. However, individual enzyme abundances varied among the three genotypes across the growth stages. For instance, *AGPas_L1* accumulated more at 12 or 15 DAA in all genotypes. *AGPas_S1* was more abundant at 7 DAA in *Cameor* and *PI 357292*, and at 12 or 15 DAA in *PS17100006*. *AGPas_S2* showed higher accumulation at 15 DAA in *Cameor* and *PI 357292*, while *PS17100006* showed consistent low abundance across the growth stages with slightly higher value at 12 DAA. *GBSSIa* peaked early in seed growth (before 15 DAA) in all lines. *GBSSIb* was less abundant in *PI 357292*, particularly at 7 DAA compared to *Cameor*, and at 12 DAA compared to *PS17100006* (Figure 6A). For *SSII*, *PI 357292* and *PS17100006* showed higher abundance at 4 DAA, decreasing during seed development (Figure 6B). In *Cameor*, *SSII* was more abundant at 12 DAA compared to the other genotypes. *SSIII* decreased in abundance during seed growth (Figure 6C). Both round pea genotypes exhibited higher levels of *SBEI* throughout seed growth stages compared to the wrinkled pea genotype *PI 357292* (Figure 6D).

##### Amino acid and protein metabolisms

We identified 47 amino acid metabolism proteins regulated by variants of 27 pea genes (Table S6). These proteins were classified into *amino acid degradation* (16 proteins), *aspartate group amino acid biosynthesis* (13 proteins), *glutamate group amino acid biosynthesis* (nine proteins), *shikimate group amino acid biosynthesis* (seven proteins), and *serine group amino acid biosynthesis* (two proteins). While amino acids are primarily essential for protein biosynthesis, they also serve as building blocks for various biosynthesis pathways and play roles in signaling and stress responses [26, 27]. For instance, we identified amino acid degradation proteins like proline dehydrogenase, which is involved in amino acid catabolic pathways in Arabidopsis [26].

Individual proteins classified under amino acid biosynthesis showed varying abundances across each seed growth stage. For example, *most amino acid degradation* proteins (10 proteins) were more abundant at maturity compared to early seed growth stages. Three *aspartate group amino acid biosynthesis* proteins, controlled by variants of *Psat1g182280* and *Psat1g182280*, and two *serine group amino acid biosynthesis* proteins, controlled by variants of *Psat1g192400*, were found to be more abundant at 4 DAA. Additionally, one *amino acid degradation* protein (encoded by *Psat1g214320*) and one *glutamate group amino acid biosynthesis* protein (encoded by *Psat1g166680*) also accumulated more at 4 DAA.

We identified 108 proteins related to protein biosynthesis (Table S6), classified into five sub‐classes: *ribosome biogenesis* (64 proteins), *organellar translation machinery* (19 proteins), *translation initiation* (14 proteins), *aminoacyl‐tRNA synthetase activity* (six proteins), and *translation elongation* (five proteins). Among these, more than half of the *ribosome biogenesis* proteins (34 proteins) peaked at 4 DAA and decreased during seed growth, reaching their lowest levels at maturity. Most *organellar translation machinery* proteins (10 proteins) were more abundant at maturity, especially in the wrinkled pea genotypes. About half of the *translation initiation* proteins (six proteins) were more abundant in the wrinkled pea genotype, particularly at 4 or 7 DAA.

##### Lipid metabolism

A total of 45 proteins, encoded by variants of 26 pea genes, were classified under lipid metabolism according to Mercator4v.6 [20]. These proteins were further categorized into *fatty acid metabolism* (22 proteins), *sphingolipid metabolism* (six proteins), *cytoplasmic lipid droplet‐associated activities* (five proteins), *lipid trafficking* (five proteins), *glycerolipid metabolism* (four proteins), and *plastoglobule‐associated activities* (three proteins) (Table S6). Most of these proteins accumulated at 12 DAA, 15 DAA, or maturity. For example, approximately 26.7%, 42.2%, and 44.4% of these proteins showed higher accumulation at maturity in *Cameor*, *PI 357292*, and *PS17100006*, respectively.

#### 3.3.3 Storage proteins and protease inhibitors

**FIGURE 7:**
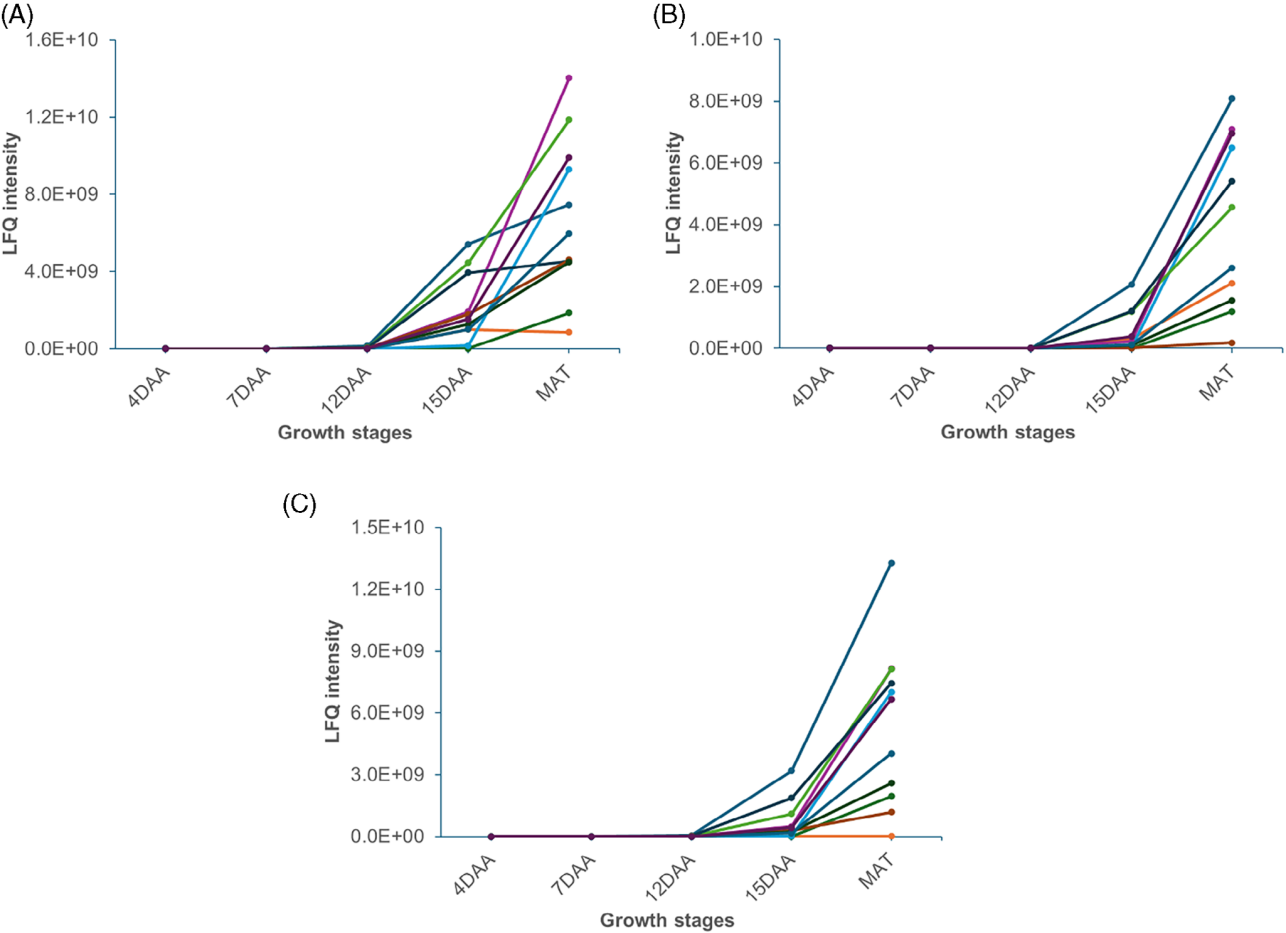
Relative abundance of storage proteins for (A) *Cameor*, (B) *PI 357292*, and (C) *PS17100006*.

Kreplak et al. [9] reported a total of 40 storage protein genes (legumin, vicilin, convicilin, and albumin) in peas. Fourteen of these storage protein genes were detected in our study, and all showed increased abundance during seed development across all three entries, particularly starting from 12 DAA, with the highest abundance observed at maturity (Figure 7).

*Legumin and* one form of *albumin* protein *(PA2)* showed higher abundance, particularly at maturity, in round peas compared to wrinkled peas (Table S6). Conversely, the wrinkled pea genotype exhibited higher levels of *vicilin* compared to the round pea genotypes. For *convicilin*, the high‐protein line (*Cameor*) accumulated more at maturity compared to the low‐protein round pea genotype (*PS17100006*) and the wrinkled pea genotype (*PI 357292*).

Protease inhibitors, including *PR6 protease inhibitor*, *kunitz protease inhibitor*, and *cystatin protease inhibitor* encoded by *Psat1g089000.1*, *Psat1g122280.1*, and *Psat1g175600.1*, respectively, increased during seed development and reached their highest abundance at maturity (Table S1). Plant protease inhibitors are primarily present in storage organs such as seeds, where they function as anti‐metabolic proteins that impede the digestion process of insects and phytopathogenic microorganisms, hence offering protection against these entities [28, 29, 30].

#### 3.3.4 Proteins classified as enzymes

The Mercator4v.6 [20] functional classification identified a significant number of enzymes (141 proteins), encoded by variants of 87 pea genes (Table S6). These proteins were further categorized into six subclasses: *oxidoreductases* (64 proteins), *transferases* (28 proteins), *hydrolases* (41 proteins), *isomerases* (four proteins), *lyases* (two proteins), and *ligases* (two proteins). Approximately 75% of *isomerases*, 56.5% of *transferases*, 47.8% of *hydrolases*, and 31.3% of *oxidoreductases* showed higher accumulation at maturity. *Lyases* exhibited higher accumulation at 15 DAA or maturity, while all *ligases* showed higher accumulation at 12 DAA or earlier growth stages.

### 3.4 Differentially abundant proteins (DAPs) in pair‐wise comparisons

For each pea genotype, 10 pair‐wise comparisons among the growth stages can be performed. However, as the time‐points were clustered into three groups (Figure 5C), we selected one representative time‐point from each group (7 DAA, 15 DAA, and maturity) for pair‐wise comparisons. Volcano plots illustrating these comparisons for the three pea genotypes are shown in Figure S2, and a summary of the results is provided in Table 1.

**TABLE 1:** Number of differentially abundant (DAPs) between pair of time‐points in the three entries.

Pea genotypes	Type	Comparisons	Upregulated	Downregulated	Total
		15 vs. 7 DAA	366	532	898
*Cameor*	Round	MAT vs. 7 DAA	499	751	1250
		MAT vs. 15 DAA	276	383	659
		15 vs. 7 DAA	369	415	784
*PI 357292*	Wrinkled	MAT vs. 7 DAA	705	784	1489
		MAT vs. 15 DAA	666	695	1361
		15 vs. 7 DAA	146	101	247
*PS17100006*	Round	MAT vs. 7 DAA	349	561	910
		MAT vs. 15 DAA	235	387	622

Our analysis revealed that the number of DAPs increased as the intervals between sampling time‐points widened (Table 1). For example, in *Cameor*, 1250 proteins were differentially abundant between 7 DAA and maturity, while 659 proteins showed differential abundance between 15 DAA and maturity. Generally, most DAPs were downregulated across the comparisons, except for the 15 versus 7 DAA comparison in *PS17100006*, where an opposite trend was observed.

Across all pair‐wise comparisons, a total of 2925 cases of DAPs were identified and linked to functional annotations (Table S7). Of these, 1983 proteins were downregulated and 942 were upregulated. Notably, most pair‐wise comparisons for proteins involved in specific biological processes showed a high percentage of downregulation. These include *chromatin organization* (98.5%), *coenzyme metabolism* (97.6%), *protein translocation* (93.5%), *cell division* (92.9%), *solute transport* (91.2%), *protein biosynthesis* (85.7%), *secondary metabolism* (85.4%), *phytohormone action* (84.2%), and *photosynthesis* (82.7%). Conversely, proteins associated with *nucleotide metabolism* (71.4%), *RNA biosynthesis* (72.7%), *polyamine metabolism* (63.2%), and *redox homeostasis* (60.0%) were predominantly upregulated.

Seed reserve accumulation is crucial during seed development. Yu et al. [15] identified 26 genes encoding proteins involved in *starch biosynthesis*. In our study, we found 45 cases of DAPs related to starch biosynthesis enzymes across the three pea genotypes at the three time‐points (7 DAA, 15 DAA, and maturity). Of these, 38 were downregulated, and seven were upregulated (Table S7). For instance, in the *Cameor*, comparisons between maturity and 15 DAA revealed that proteins associated with five starch biosynthesis genes (*SuSy1*, *SuSy3*, *AGPas_S2*, *AGPas_L1*, and *PGM*) were downregulated. In the comparison between maturity and 7 DAA, proteins from seven starch biosynthesis genes (*GBSSIb*, *GBSSIa*, *SuSy1*, *SuSy3*, *ISA3*, *AGPas_L1*, and *PGM*) were downregulated. In the 15 versus 7 DAA comparison, *GBSSIb*, *GBSSIa*, and *ISA3* were downregulated, while *AGPas_S2*, *UGPase*, *SBEI*, and *AGPas_L1* were upregulated. Similarly, for the other two pea genotypes, most comparisons showed a trend toward downregulation for starch biosynthesis enzymes. As anticipated, storage proteins were consistently upregulated across all pair‐wise comparisons for all three pea genotypes (Table S7).

Another crucial process in seed development is cell division. We identified 70 DAPs related to cell division across the three pea genotypes and time‐points (7 DAA, 15 DAA, and maturity) through pair‐wise comparisons (Table S7). Among these, all 59 cases related to *cytokinesis* and all six cases related to *mitochondrion/peroxisome binary fission* were downregulated. Conversely, five pair‐wise comparisons for *cell cycle organization* showed upregulation. Specifically, the *Chromosome Passenger Complex (CPC)* protein encoded by *Psat2g132080*, involved in cell cycle organization, was upregulated in five of the nine pair‐wise comparisons: 15 versus 7 DAA in *Cameor* and *PI 357292*, maturity versus 15 DAA in *Cameor* and *PS1710006*, and maturity versus 7 DAA in *PS1710006*.

## 4 DISCUSSION AND CONCLUSION

We conducted label‐free proteomics to analyze the proteome profiles of three pea genotypes (two round and one wrinkled) during seed development. Pea seeds typically reach maturity approximately 37 DAA. Our study focused on early seed development (up to 15 DAA), with four sampling points in this period, and one sampling time‐point at maturity. This design highlights protein profiles in the early seed growth period but does not capture changes throughout the entire seed development. Cluster analysis grouped 4 and 7 DAA together, and 12 and 15 DAA in another group, suggesting that fewer sampling points could suffice within these clusters, while additional points from later stages could be beneficial. Thus, two sampling strategies are recommended: (1) concentrate sampling within a specific stage, such as early development; or (2) sample at regular intervals (e.g., every 7 days) to cover the full range of seed development period.

The two round pea genotypes showed similar growth patterns, with a steady increase in FW until 27–32 DAA, followed by a sharp decline. Their DW rose rapidly from 15 to 27 DAA. In contrast, the growth pattern of the wrinkled pea genotype was distinct. Both round and wrinkled peas had comparable FW gains until 27 DAA, but wrinkled peas experienced a rapid increase in FW due to higher moisture content between 27 and 34 DAA.

The wrinkled peas had much lower levels of starch branching enzyme I (SBEI), aligning with previous findings that wrinkled peas have defective SBEI and SBEII [14, 16, 31]. The wrinkled peas also contained less starch and lower *legumin* protein levels but had a higher amylose/amylopectin ratio and increased sucrose and lipid levels [16, 31]. Our study confirmed lower *legumin* accumulation in wrinkled peas compared to round peas. Elevated sucrose in wrinkled peas increases osmotic pressure, leading to greater water uptake, increased cell size, and higher FW. However, as the seeds mature, they lose water, resulting in their wrinkled phenotype.

Seeds contain essential components that protect and nourish the embryo, providing energy during germination until the seedling can photosynthesize [32]. Seed development involves a complex gene interplay. Early stages are marked by high mitotic activity, determining seed size. Later stages focus on accumulating reserves like starch and protein, ending with desiccation and dormancy at maturity [3, 4, 33, 34, 35, 36]. The shift from active cell division to reserve accumulation is triggered by a change from a hexose‐rich to a sucrose‐rich state [3]. During this phase, seeds also enhance their photosynthetic activity. Our study found several proteins with differential abundance related to cell division, photosynthesis, and storage product biosynthesis across the seed development period.

During early seed development, cell division dominates, leading to higher abundance of cell division‐related proteins. Our findings confirmed this, showing that these proteins were mostly downregulated in later stages. The cell cycle has four phases [4, 37]: (1) G1 phase (cell proliferation and organelle duplication), (2) S phase (complete nuclear DNA synthesis), (3) G2 phase (cell growth and formation of proteins and organelles), and (4) M phase (division of copied DNA and cytoplasm). One of the DAPs identified was a microtubule‐associated protein, which is known to segregate chromosomes during mitosis and promote cell proliferation and axial growth in *Arabidopsis* [38, 39, 40].

Photosynthesis produces sucrose, the primary sugar transported in the plant phloem [24]. While seeds are crucial for reproduction and nutrient storage, they can also photosynthesize [41]. Seed photosynthesis enhances biosynthetic flux by increasing internal oxygen and energy supply during seed development [23, 42, 43]. In our study, photosynthesis‐related proteins were mostly downregulated as seeds grew. This aligns with previous findings that seed photosynthesis is prominent early in seed development when seeds are still green [23, 43].

Amino acids are synthesized via several pathways: the shikimate pathway for aromatic amino acids such as phenylalanine, tyrosine, and tryptophan [44, 45], the aspartate pathway for lysine, methionine, isoleucine, and threonine [45, 46, 47], and the pyruvate pathway for valine, leucine, and isoleucine [45]. In our study, 47 proteins involved in amino acid biosynthesis were identified, with higher abundance observed during early seed development compared to mature seeds. Free amino acids primarily contribute to seed storage protein synthesis. Kreplak et al. [9] identified 40 storage protein genes in peas, with 14 detected in our proteomic study. These storage proteins increased in abundance throughout seed development, peaking at maturity, indicating their role as markers of seed maturity [33]. Round peas showed higher levels of *legumin* and *Albumin* isoform PA2, while wrinkled peas had higher levels of *vicilin*. Previous studies noted elevated *legumin* levels in round peas [31, 48, 49]. In addition, wrinkled peas had a higher *vicilin/legumin* ratio than round peas, according to Gueguen and Barbot [50].

In summary, our study identified key proteins involved in cell division, carbohydrate metabolism, and amino acid biosynthesis during seed development and maturity. These findings highlight the crucial role of these proteins in seed formation and growth. Our dataset serves as a valuable resource for researchers and breeding programs aiming to enhance nutritional content, seed size, germination, and pathogen resistance. By linking these proteins to a *Cameor*‐based reference genome [9], our study opens avenues for deeper investigations into individual proteins, advancing our knowledge of seed development and facilitating their practical application in crop improvement.

## AUTHOR CONTRIBUTIONS

Sintayehu D. Daba: Contributed to conceptualizing the project, collecting the samples and phenotypic data, data analysis, and preparing and reviewing the manuscript. Punyatoya Panda: Contributed to proteomic sample preparation, data analysis, and reviewing the manuscript. Uma K. Aryal: Contributed to generating proteomic data, data analysis, and reviewing the manuscript. Alecia M. Kiszonas: Contributed to reviewing the manuscript. Sean M. Finnie: Contributed to reviewing the manuscript. Rebecca J. McGee: Contributed to conceptualizing the project, providing financial support for the project, and reviewing the manuscript.

## CONFLICT OF INTEREST STATEMENT

The authors declare no conflict of interest.

## Supporting information

Supporting Information

Supporting Information

## Data Availability

All the LC‐MS/MS raw data files related to this study are deposited in Massive data repository (https://massive.ucsd.edu) with submission ID: MSV000095262 and are publicly available.
