# Supplementary material for: Proteomics analysis of round and wrinkled pea (Pisum sativum L.) seeds during different development periods
Source: Proteomics. 2024 Oct 30;25(3):2300363. doi: 10.1002/pmic.202300363 (PMC11794676; doi:10.1002/pmic.202300363)
Supplement: Supplementary file 1 — Supporting Information [file PMIC-25-2300363-s002.docx]

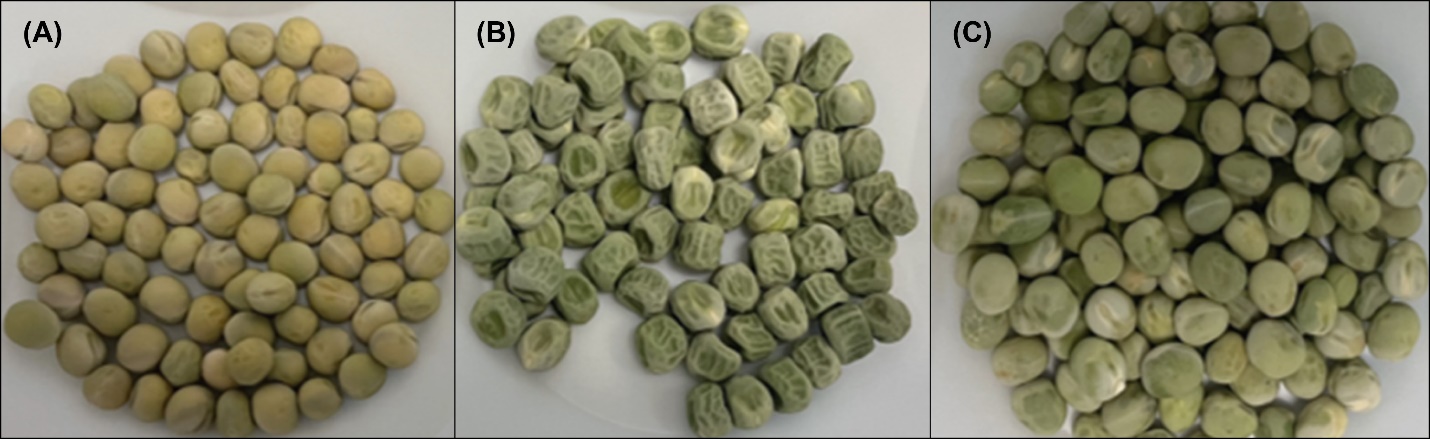
**Figure S1**. Pictures of seeds for (**A**) *Cameor*, (**B**) *PI 357292*, and (**C**) *PS1710006*.


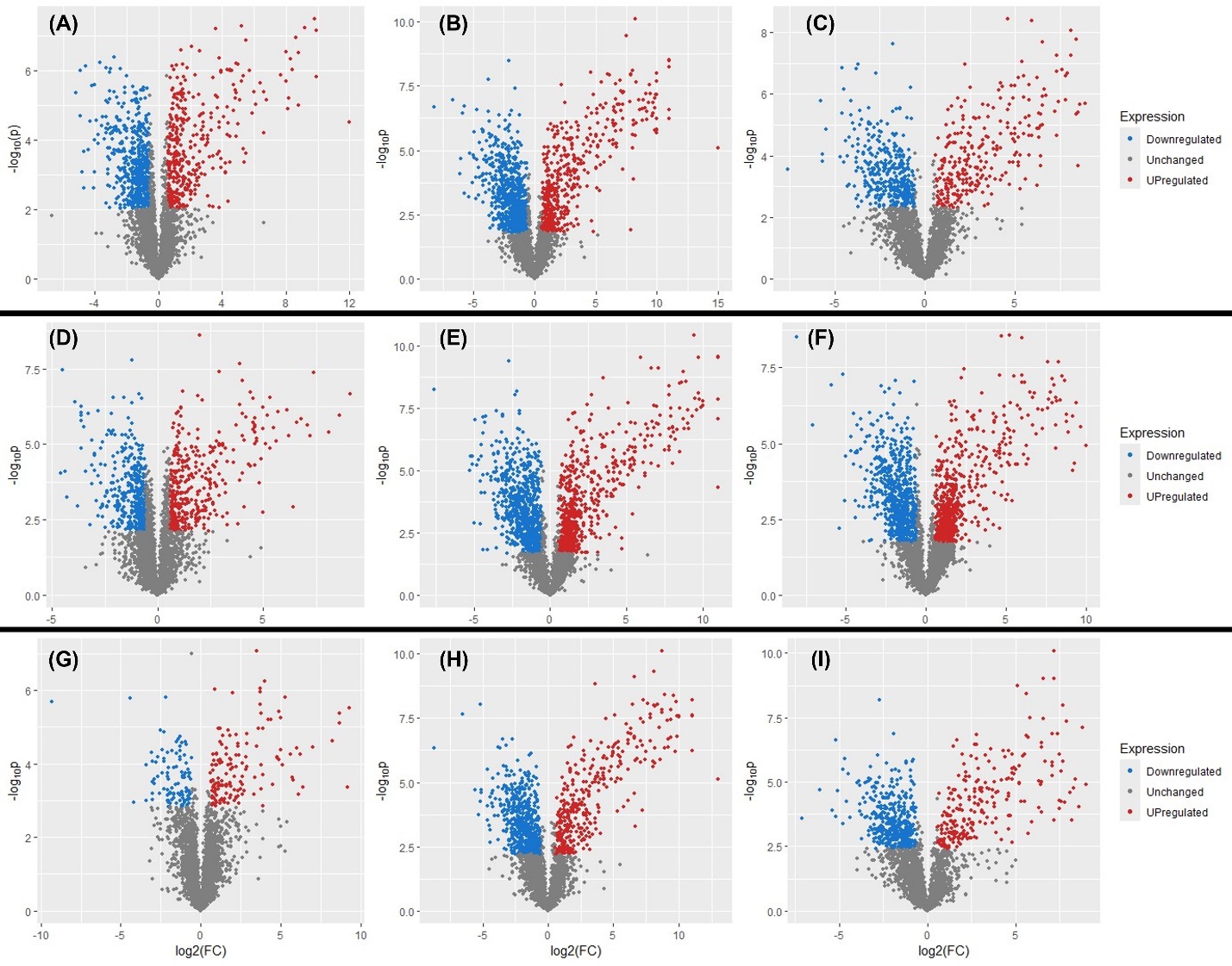
**Figure S2**. Volcano plots in pair-wise comparisons among growth stages for *Cameor* [(**A**) 15DAA vs 7DAA, (**B**) maturity vs 7DAA, and (**C**) maturity vs 15DAA], for *PI 357292* [(**D**) 15DAA vs 7DAA, (**E**) maturity vs 7DAA, and (**F**) maturity vs 15DAA], and for *PS17100006* [(**G**) 15DAA vs 7DAA, (**H**) maturity vs 7DAA, and (**I**) maturity vs 15DAA].
